# A point mutation in the DNA-binding domain of HPV-2 E2 protein increases its DNA-binding capacity and reverses its transcriptional regulatory activity on the viral early promoter

**DOI:** 10.1186/1471-2199-13-5

**Published:** 2012-02-15

**Authors:** Chen Gao, Ming-Ming Pan, Yan-Jun Lei, Li-Qing Tian, Hui-Ying Jiang, Xiao-Li Li, Qi Shi, Chan Tian, Yu-Kang Yuan, Gui-Xiang Fan, Xiao-Ping Dong

**Affiliations:** 1 State Key Laboratory for Infectious Disease Prevention and Control, National Institute for Viral Disease Control and Prevention, Chinese Center for Disease Control and Prevention, Changbai Rd 155, Beijing 102206, People's Republic of China; 2 School of Medicine, Xi'an Jiao-Tong University, Xi'an 710061, People's Republic of China; 3 Center for Computational and Systems Biology, Institute of Biophysics, Chinese Academy of Science, Beijing 100101, People's Republic of China; 4 Chinese Academy of Sciences Key Laboratory of Pathogenic Microbiology and Immunology, Institute of Microbiology, Chinese Academy of Sciences, Beijing 100101, People's Republic of China

**Keywords:** HPV-2, E2, DNA-binding, Transcriptional regulation, Promoter

## Abstract

**Background:**

The human papillomavirus (HPV) E2 protein is a multifunctional DNA-binding protein. The transcriptional activity of HPV E2 is mediated by binding to its specific binding sites in the upstream regulatory region of the HPV genomes. Previously we reported a HPV-2 variant from a verrucae vulgaris patient with huge extensive clustered cutaneous, which have five point mutations in its E2 ORF, L118S, S235P, Y287H, S293R and A338V. Under the control of HPV-2 LCR, co-expression of the mutated HPV E2 induced an increased activity on the viral early promoter. In the present study, a series of mammalian expression plasmids encoding E2 proteins with one to five amino acid (aa) substitutions for these mutations were constructed and transfected into HeLa, C33A and SiHa cells.

**Results:**

CAT expression assays indicated that the enhanced promoter activity was due to the co-expressions of the E2 constructs containing A338V mutation within the DNA-binding domain. Western blots analysis demonstrated that the transiently transfected E2 expressing plasmids, regardless of prototype or the A338V mutant, were continuously expressed in the cells. To study the effect of E2 mutations on its DNA-binding activity, a serial of recombinant E2 proteins with various lengths were expressed and purified. Electrophoresis mobility shift assays (EMSA) showed that the binding affinity of E2 protein with A338V mutation to both an artificial probe with two E2 binding sites or HPV-2 and HPV-16 promoter-proximal LCR sequences were significantly stronger than that of the HPV-2 prototype E2. Furthermore, co-expression of the construct containing A338V mutant exhibited increased activities on heterologous HPV-16 early promoter P97 than that of prototype E2.

**Conclusions:**

These results suggest that the mutation from Ala to Val at aa 338 is critical for E2 DNA-binding and its transcriptional regulation.

## Background

Human papillomaviruses (HPVs) are small, double-stranded DNA viruses that infect the mucosal epithelial tissues of anogenital tract, oral cavity and upper alimentary tract, as well as cutaneous epithelial tissues of hands, feet and trunks. HPVs have been grouped into cutaneous type that causes cutaneous warts and epidermodysplasia verruciformis, and mucosal type that predominantly induces benign and malignant lesions of the genital tract, in which HPV-2 has been frequently associated with verrucae vulgaris [1]. HPV-2 genome is composed of eight open reading frames (ORFs) encoding the regulatory proteins essential for completion of the viral life cycle and the structural components of the virion, respectively [2].

HPVs' E2 proteins are believed to control the transcriptions of viral genes through binding to the specific sites in viral DNA, multiple copies of which are found in the viral upstream regulatory regions (URRs) [3]. The HPV E2 protein can function as either a repressor or an activator of the early gene transcription, depending on the location of E2 binding sites in the viral regulatory region as previously demonstrated for genital HPVs [4]. The structure of the E2 protein resembles a typical transcription factor, with an amino-terminal transcriptional activation domain (TAD) and a carboxyl-terminal DNA-binding/dimerization domain (DBD), separated by a variable hinge region [2]. The E2 protein exists in solution and binds to the target DNA as a dimmer. The HPV-16 E2-DBD forms a dimeric β-barrel, with each subunit contributing an anti-parallel 4-stranded β-sheet "half-barrel" [5]. Several studies showed that E2 acts as a transactivator at low concentrations, while as a repressor at high concentration. Recently, it has been reported that the locations of E2 binding sites are important for transcriptional repression, independent of binding affinities [6].

Besides being a transcriptional regulator in the life-cycle of virus, E2 protein is believed to play an important role in the carcinogenesis of HPV-associated cancers. The HPV genome can exist in the malignant cells in two forms, integrated into the host chromosome or episomal DNA. The majority of HPV-associated cancers, especially cervical carcinoma, contain integrate HPV DNA [7]. Usually, integration of viral genome into host chromosome results in disruptions of E2 and E1 ORFs, leading to an increased transcription from the viral early promoter and elevated expression of viral oncogenes E6 and E7 [7,8]. About 15-20% HPV-positive cervical cancers contain intact HPV genomes in extrachromosomal state. Various point mutations or deletions in the HPV genome were reported to be related to the viral oncogenesis potential, e.g. in the long control region (LCR) [9], E2 and E1 ORFs [10]. Previously we reported a verrucae vulgaris patient with huge extensive clustered cutaneous who was confirmed to be infected by a HPV-2 variant [11,12]. Several point mutations were detected in the LCR of this HPV-2 variant that lead to an increased promoter activity. In addition, five point mutations were found within the E2 ORF. Expression of the E2 mutant exhibited increased activities on the viral early promoter as compared with the prototype E2 [13].

In order to gain insight into the potential influences of these mutations within E2, we constructed a series of mammalian and prokaryotic expressing plasmids encoding E2 proteins with one to five amino acid (aa) substitutions. Upon co-transfection with a CAT reporter under the control of HPV-2 LCR, the E2 construct containing the A338V mutation within the DNA-binding domain functioned as a transactional activator instead of repressor. Electrophoretic mobility shift assays (EMSA) demonstrated that the ability of E2 protein with A338V mutation bind a double-stranded DNA sequence containing two E2 binding sites is markedly stronger than HPV-2 prototype E2. The binding affinity of the E2 A338V mutant for the promoter-proximal LCR sequences of HPV-2 and HPV-16 were also significantly increased. Structural analyses indicated that the mutation A338V located in the region of beta barrel. These results imply that the mutation A338V is critical for the E2 DNA-binding and promoter regulation.

## Results

### The A338V mutation within the HPV-2 E2 DNA-binding domain is critical for E2 transcriptional regulation activity on the HPV-2 early promoter

**Figure 1:**
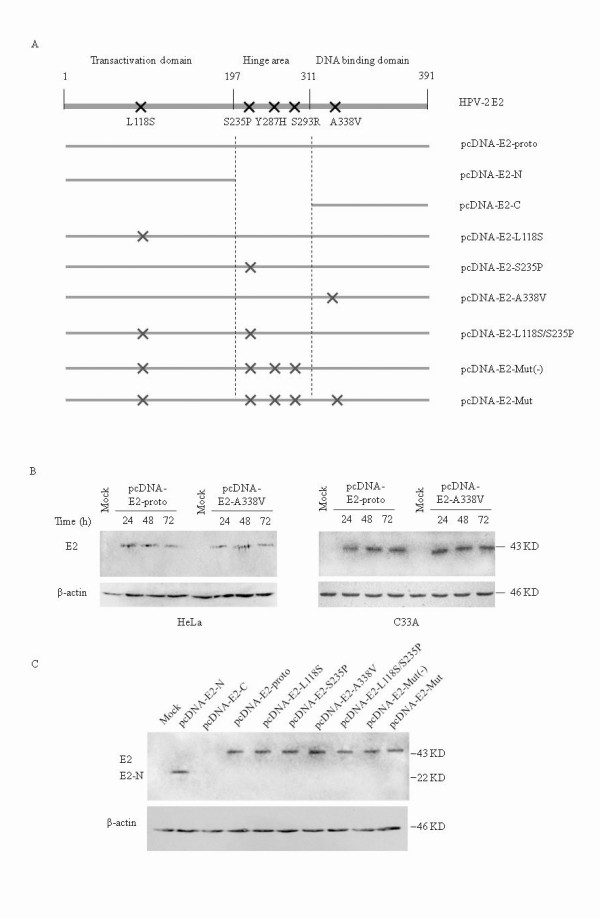
**Expressions of various HPV-2 E2 proteins in the cultured cells**. **A**. Schematic structures of the full-length E2 and various mutated E2. The black crosses indicate the amino acid exchanges of these mutants. **B**. HeLa (left panel) or C33A (right panel) cells were either mock transfected or transfected with 500 ng of pcDNA-E2-proto or pcDNA-E2-A338V as indicated. Cells were harvested 24, 48 or 72 h post-transfection. The prepared cells extracts were separated by 15% SDS-PAGE, transferred to nitrocellulose membrane and probed with E2 or β-actin antibody as indicated. Exposition time was 5 min for E2 and 2 min for β-actin. The blot shown is a representative experiment among three experiments. **C**. 500 ng of various E2 constructs were co-transfected with 2 μg CAT reporter plasmid pCAT-LCR into HeLa cells, respectively. Cells were harvested 48 h post-transfection. The blot shown is a representative experiment among three experiments. The blots of E2, E2-N and β-actin are indicated on the left and relative molecular weights are arrowed on right

To assess the effect of the point mutations within E2 on its transcriptional activity, a series of HPV-2 E2 mammalian expressing plasmids were constructed. These include the point mutations within the E2 transactivation, hinge and DNA-binding regions. In addition, two plasmids expressing truncated E2 proteins were also generated (Figure 1A). To detect expression of HPV-2 E2 from the transiently transfected plasmids in the cultured cells, HeLa and C33A cells transfected with pcDNA-E2-proto and pcDNA-E2-A338V were harvested 24, 48 and 72 h post-transfection, respectively. The presences of HPV E2 proteins in cell lysates were confirmed by Western blot with a HPV-2 E2 specific monoclonal antibody (mAb) prepared with a full-length recombinant HPV-2 E2 protein as immunogen, which recognized the segments of N-terminus and hinge region of E2 protein (unpublished) (Figure 1B). In addition, E2 expressing in HeLa cells were also evaluated by Western blots after co-transfection of the HPV-2 E2 plasmids with the pCAT-LCR (L) reporter plasmid. As shown in Figure 1C, the full-length E2 (43 KD) and N-terminal E2 (22 KD) were detected, whereas no signal was observed for the C-terminal E2.

**Figure 2:**
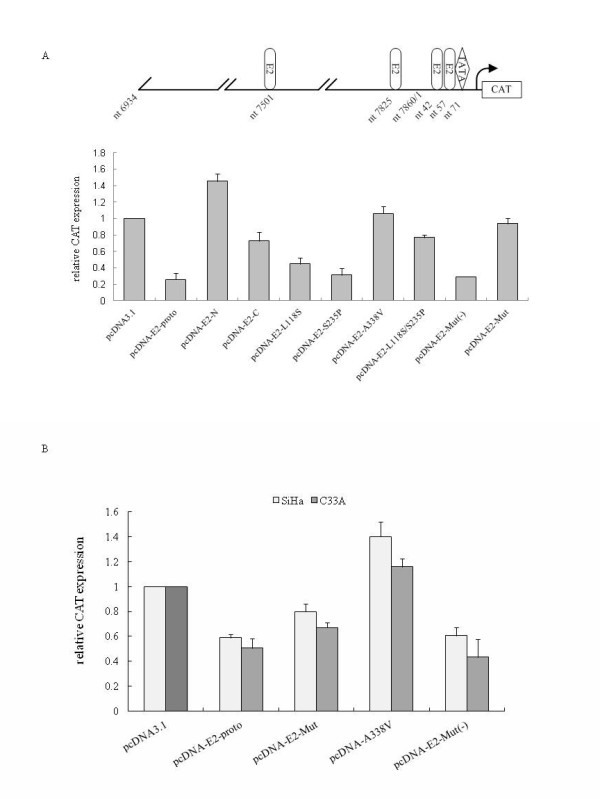
**The influences of various HPV-2 E2 constructs on the promoter activity under control of the LCR of HPV-2**. **A**. The relative CAT expressions co-transfected with different E2 expressing plasmids in HeLa cells. The schematic structure of HPV-2-LCR cloned in the CAT reporter plasmid pBL-CAT6 is shown above. HPV-2-LCR covers the sequences from nt 6934 to 134. The positions of TATA box and four potential E2 binding sites are indicated with the starting nucleotide (nt) below. HeLa cells were transfected with 2 μg of plasmid pCAT-LCR and 500 ng of either mock plasmid (pcDNA3.1) or the plasmids for various E2 constructs (as indicated). 1 μg pCMV-β-galactosidase was transfected as internal control. Cells were harvested 48 h after transfection. The expressions of CAT and β-galactosidase were determined. The CAT expression each preparation was normalized with its β-galactosidase value. The relative CAT expressions are averaged from at least three independent experiments and presented relative to that of pcDNA3.1. Data are represented as mean ± SEM. **B**. The relative CAT expressions co-transfected with different E2 expressing plasmids in C33A and SiHa cells. C33A and SiHa cells were transfected with 2 μg of plasmid pCAT-LCR and 500 ng of either mock plasmid (pcDNA3.1) or the plasmids for various E2 constructs (as indicated). 1 μg pCMV-β-galactosidase was transfected as an internal control. The relative CAT expressions are averaged from at least three independent experiments in C33A and SiHa cells and presented relative to that of pcDNA3.1. Data are represented as mean ± SEM

Under our experimental condition, transfection of the blank CAT reporter vector (pBL-CAT6) did not induce detectable CAT expression (data not shown). Consistent with previous observations in HeLa cells containing HPV-18 genome [13], co-transfection of pcDNA-E2-proto significantly reduced the HPV-2 LCR driven CAT expression (Figure 2A, pcDNA-E2-proto), whereas co-transfection of pcDNA-E2-Mut significantly increased the CAT expression (pcDNA-E2-Mut). Co-transfected with the plasmids encoding N- (pcDNA-E2-N) and C-terminal (pcDNA-E2-C) E2 resulted in significantly higher CAT expressions than that with the plasmid encoding full-length prototype E2. Transfection of the plasmids expressing single point mutation within the transactivation domain (Figure 2A, pcDNA-E2-L118S) and the hinge region (Figure 2A, pcDNA-E2-S235P) resulted in comparable CAT expressions as that of prototype E2, while pcDNA-E2-L118S/S235P induced a relatively higher CAT expression. Interestingly, transient transfection of pcDNA-E2-A338V containing a single point mutation in the E2 DNA-binding domain led to a significantly increased CAT expression that was even slightly higher than that of pcDNA-E2-Mut. In contrast, transfection of pcDNA-E2-Mut (-) containing all four point mutations but A338V caused a significant repression of CAT expression that was comparable with pcDNA-E2-proto. These results demonstrate that the A338V mutation within the DNA-binding domain is essential for the E2 repression activity.

Next, we examined the transcriptional activity of E2 with A338V mutation the HPV-negative cervical cancer cell lines C33A and the HPV-16 genome-containing SiHa cells. Consistent with the results in HeLa cells, under the control of HPV-2 LCR, co-expression of pcDNA-E2-proto and pcDNA-E2-Mut (-) led to obviously low CAT expressions compared with mock, whereas co-expressions of pcDNA-E2-Mut and pcDNA-E2-A338V caused high CAT expressions (Figure 2B). Notably, in pcDNA-E2-A338V-transfected cells, the relative CAT expression was higher than that of mock. These results imply that the transcriptional repression activity of E2 mutant A338V is independent of the endogenous HPV genome.

### The A338V E2 mutant increased the binding capacity to the DNA sequences containing conservative E2 binding sites in vitro

**Figure 3:**
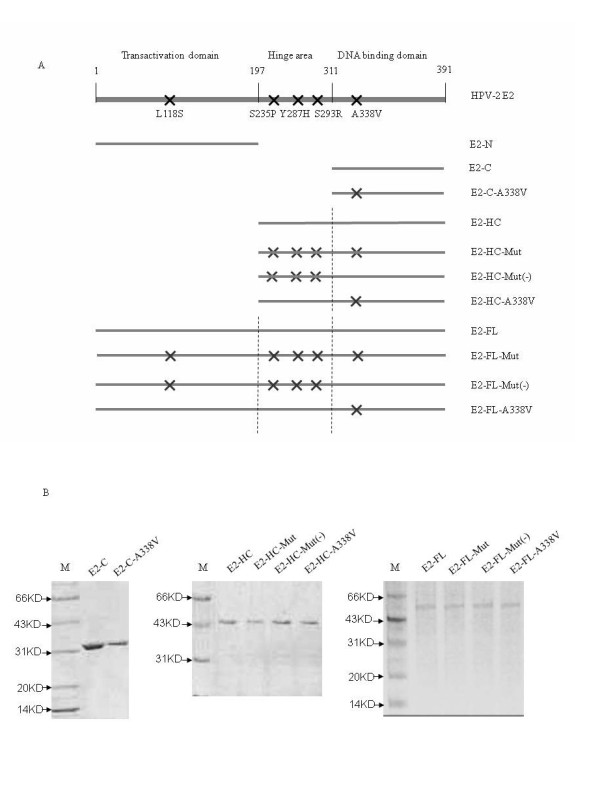
**Expressions and purifications of various recombinant HPV-2 E2 proteins**. **A**. Schematic structures of the full-length E2 and various mutated E2. The mutations are marked by the black crosses. **B**. 15% SDS-PAGE assays of various purified recombinant E2 proteins. Full length and various mutated E2 protein with GST tag were expressed in *E. coli *BL21 and purified with Glutathione sepharose 4B Agarose. 0.5 μg of each purified E2 protein was loaded on 15% SDS-PAGE and stained by coomassie brilliant blue. Each purified protein is indicated on the tops of the graphs. M: Protein molecular markers. The molecular sizes are shown on the left

To explore the mechanism for derepression on the HPV-2 promoter activity caused by the mutation A338V, a serial of recombinant E2 proteins were expressed and purified in *E. coli*. Figure 3A summarizes the E2 proteins in different contexts, including one construct of E2 transactivation domain, two constructs of E2 DNA-binding region, four constructs of E2 hinge region and DNA-binding domain and four constructs of full-length E2. All proteins were expressed in soluble form as GST-fusions (Figure 3B).

**Figure 4:**
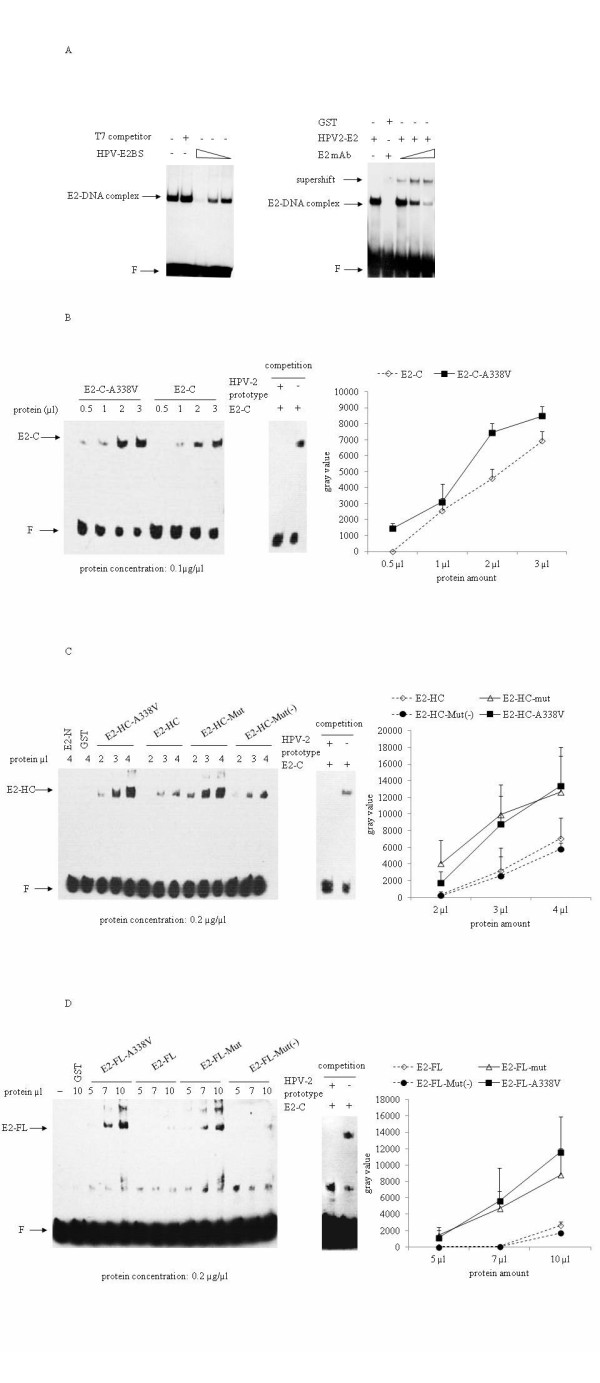
**Evaluations for DNA-binding capacities of various HPV-2 E2 proteins with oligo HPV-E2BS by EMSA**. **A**. Specificity assays of the molecular binding of oligo HPV-E2BS with recombinant HPV-2 E2. Left panel: Competition assays. 250 fM biotin-labeled oligonucleotide probe HPV-E2BS were mixed with 1 μg recombinant protein HPV-2 E2-FL, competed with 50-, 100-, and 500-fold excesses of homologous oligo (HPV-E2BS) and 500-fold excesses of heterologous oligo (T7). Oligonucleotide T7 represents *E. coli *T7 promoter-specific double-stranded sequences (5'-TCGATAATACGACTCACTATAGGGAGAAGATC-3'). Right panel: Supershift assay with mAb anti-HPV-2 E2. 7 μg recombinant HPV-2 E2-FL was incubated with 1 μl mAb against HPV-E2 at RT, prior to mixing with 250 fM biotin-labeled probe HPV-E2BS. The E2-DNA complexes and the supershifts are indicated by arrows. F: free probe. **B**. EMSA of various constructs of E2-C. **C**. EMSA of various constructs of E2-HC. **D**. EMSA of various constructs of E2-FL. 250 fM biotin-labeled probe HPV-E2BS were mixed with different amounts of various E2 proteins. The concentrations of various E2 protein were indicated in the bottom of the graphs. The protein-DNA complexes were separated in 6.5% PAGE gels (Left) indicated by arrows marked E2-FL, E2-HC and E2-C, respectively. The competition assays were performed in the presences of 500-fold excess of unlabeled probe HPV-E2BS prototype (Middle). F: free probe. The binding capacities of various E2 proteins (Figure 4B, C and D) were evaluated by densitometric quantification of the signal of each complex with computer-assisted software Image TotalTech. The average values are calculated from three independent tests and presented with as mean ± SD (Right)

Using the biotin-labeled double-stranded oligo HPV-E2BS containing two E2 protein binding sites (E2BS), the DNA-binding activities of different expressed E2 proteins were evaluated by EMSA. The specificity of oligo HPV-E2BS for HPV E2 protein was first evaluated by competition experiments with homologous or heterologous unlabeled oligos. Compared with the clear DNA-protein complex formation in the mixture of HPV-2 E2 and oligo HPV-E2BS, addition of the excessive cold homologous oligo instead of the heterologous oligo T7 (Figure 4A, left panel). To get more evidences on the specificity of the binding of oligo HPV-E2BS with E2 protein, the recombinant HPV-2 E2 was incubated with a mAb against HPV-2 E2 prior to EMSA. Along with the reductions of the signals of the DNA-E2 complexes in the presence of mAb anti-E2, obvious supershifts were detected (Figure 4A, right panel). These results indicate the interaction between oligo HPV-E2BS and E2 protein is specific.

In the context of E2 binding domain, both prototype E2 and the A338V mutant E2 formed protein-DNA complexes with the probes in a dose-dependent manner. Interestingly, the binding activity of E2 mutant was significant stronger than that of prototype E2 (Figure 4B). To confirm this phenomenon, four E2 proteins covering E2 hinge region and DNA-binding region were employed into EMSA. Figure 4C showed that the DNA-binding activity of E2-HC-A338V was stronger than that of prototype E2-HC. Additionally, E2-HC-Mut with A338V and other three point mutations in hinge region showed similar DNA-binding activity as E2-HC-338 V, while E2-HC-Mut (-) with only three mutations in hinge region caused similar DNA-binding activity as prototype E2-HC. As expected, neither GST nor E2 transactivation domain (E2-N), formed a complex in EMSA. Similar manner were observed in the EMSA in the context of full-length E2 protein, in which two constructs containing the A338V mutation (E2-FL-A338V and E2-FL-Mut) formed more obvious protein-DNA complexes than the other two constructs without the A338V mutation (E2-FL and E2-FL-Mut (-)), regardless of having the point mutations in the transactivation domain and hinge area (Figure 4D). These results highly suggest that the substitution of Ala to Val at residual 338 in HPV-2 E2 protein influences critically its DNA-binding affinity.

### E2 DNA-binding affinities were influenced by the length of the E2 peptides

**Figure 5:**
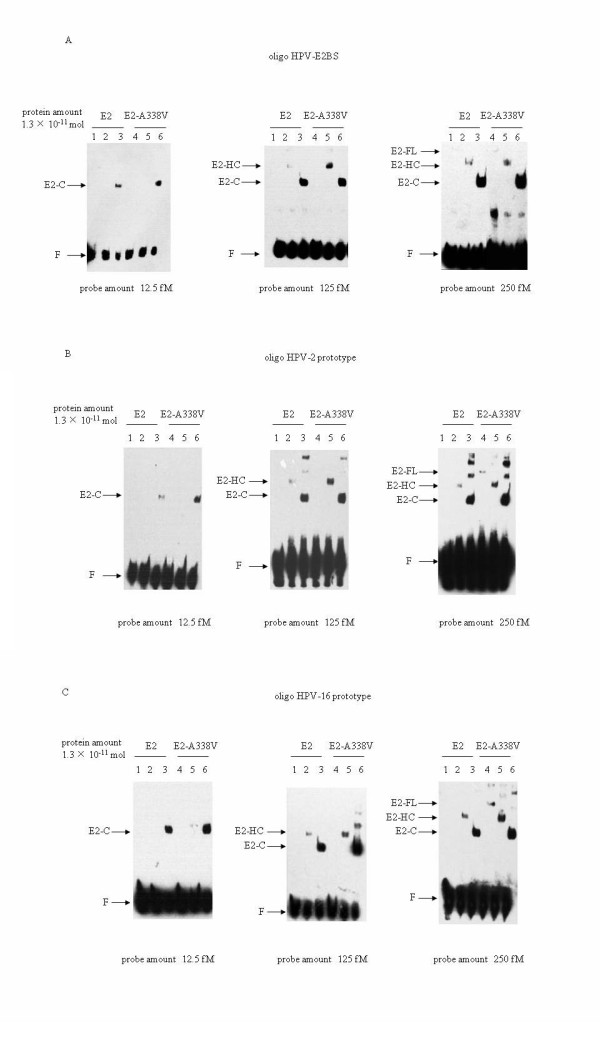
**Comparative analyses of DNA-binding activities between the HPV-2 prototype E2 (represented as E2) and mutated E2 at the position of residual 338 (represented as E2-A338V)**. **A**: oligo HPV-E2BS; **B**: oligo HPV-2 prototype; **C**: oligo HPV-16 prototype. Equally molar amounts (1.3 × 10^-11 ^mol) of E2 and E2-A338V proteins in three different constructs were mixed the individual biotin-labeled probes and the protein-DNA complexes were separated in 6.5% PAGE gels. Lane 1 and 4: E2-FL protein; Lane 2 and 5: E2-HC; Lane 3 and 6: E2-C. The probe amounts used in EMSA are indicated at the bottom of the graphs and the protein amounts are shown above the graphs. The protein-DNA complexes of the preparations are indicated by arrows marked E2-C (lane 1 and 4), E2-HC (lane 2 and 5) and E2-FL (lane 3 and 6), respectively. F: free probe

From the EMSA results shown in Figure 4, it seemed that the DNA-binding activities of E2 were also affected by the length of the peptides. To address this possibility, the same molar number of E2 proteins in three different lengths was mixed with biotin-labeled oligos. With 12.5 fM of oligos, only the E2-C construct formed detectable protein-DNA complexes (Figure 5, left panel). The protein-DNA complexes of E2-HC constructs were clearly observed when the amount of oligo was increased to 125 fM, while that of E2-C became much stronger (Figure 5, middle panel). When the amounts of oligo increased to 250 fM, the protein-DNA complexes formed by the constructs of E2-FL became visible (Figure 5, right panel). In addition, the A338V E2 formed abundant protein-DNA complex in all three lengths of E2 peptides tested and in all three concentrations of oligo. These results suggest that the transactivation domain and the hinge region of E2 play a negative role in its DNA binding affinity.

### The A338V E2 mutants increased the binding affinity to the promoter-proximal LCR sequences of HPV-2 and HPV-16

To evaluate the DNA-binding activities of E2 with A338V to the wild-type HPV sequences, the biotin-labeled double-stranded oligos derived from the sequences of prototype HPV-2 and HPV-16 LCRs, which contained two E2 protein binding sites, were mixed with equal amount of the different recombinant prototype and mutated E2 proteins and assessed in EMSA. Consistent with results shown in Figure 4, the A338V E2 mutants showed clearly stronger binding affinities to both HPV-2 (Figure 5B) and HPV-16 (5 C) oligos than the HPV-2 prototype E2, in the context of either full-length or truncated forms. No difference was observed in the binding affinity of HPV-2 E2 to the LCR sequences of homologous or heterologous HPV genotypes. These results show that A338V E2 mutant has stronger binding affinity to the promoter-proximal LCR sequences of wild-type HPVs. E2 C-terminus (E2-C) possessed much stronger binding activities to HPV-2 and HPV-16 LCR than E2-HC and E2-FL, which were coincident well with the binding tendency of different E2 in length shown in Figure 4B, C and 4D. The multiple bands at higher molecular weight position in the gels (Figure 4C, D and 5) may represent the dimmers of the E2 proteins.

Co-expressions of HPV-2 E2 mutants with A338V induced more active activity on heterologous HPV-16 early promoter P97 than the HPV-2 prototype E2.

**Figure 6:**
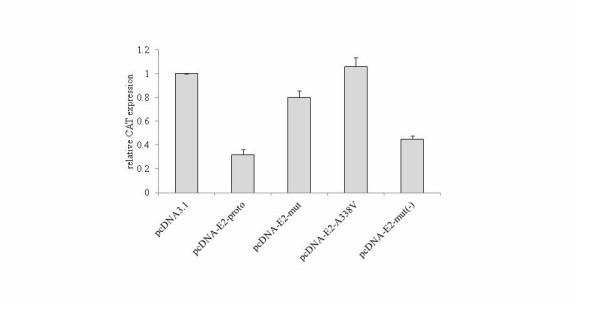
**The influences of various HPV-2 E2 constructs on the promoter activity under control of the LCR of HPV-16**. The relative CAT expression co-transfected with different E2 expressing plasmids was evaluated. HeLa cells were transfected with 2 μg of plasmid pCAT-HPV16-LCR and 500 ng of either mock plasmid (pcDNA3.1) or the plasmids for various E2 constructs (as indicated). 1 μg pCMV-β-galactosidase was transfected as internal control. Cells were harvested 48 h after transfection and the expressions of CAT and β-galactosidase were determined. The CAT expression each preparation was normalized with its β-galactosidase value. The relative CAT expressions are averaged from three independent experiments and presented relative to that of pcDNA3.1. Data are represented as mean ± SEM

In order to figure out whether the E2 mutation A338V induced similar effectiveness on viral early promoter of heterologous genotype HPV, a CAT-reporter plasmid under the control of HPV-16 LCR was co-transfected with same amount of various HPV-2 E2 expressing plasmids, including pcDNA-E2-proto, pcDNA-E2-Mut(-), pcDNA-E2-Mut and pcDNA-E2-A338V, respectively. Remarkably decreased CAT expression was observed when pcDNA-E2-proto was co-transfected (Figure 6, column 2), indicating that HPV-2 E2 was able to inhibit the activity of HPV-16 promoter P97. Similar to the observations under the control of HPV-2 LCR, expressions of either E2 with single A338V mutation (pcDNA-E2-A338V, column 4) or A338V plus other four point mutations (pcDNA-E2-Mut, column 3) resulted in significantly more CAT expressions under the control of HPV-16 LCR. As expected, transfection of pcDNA-E2-Mut (-) (column 5) with other four point mutations except A338V still maintained the same repression on P97 activity as that of pcDNA-E2-proto. These data suggest that the A338V E2 mutant may reverse its regulation activity on viral early promoters of HPVs with similar upstream constructs.

## Discussion

In this study we have provided evidence data that a naturally occurred mutation of A338V in HPV-2 E2 increases E2 DNA-binding capacity and reverses its transcriptional regulation activity on the viral early promoter. The effect of this mutation on the biological functions of E2 seems to be very critical, since the other four amino acid exchanges locate at the transactivation domain and the hinge regions of E2 have little impact. The E2 protein has the typical structure of transcriptional regulator, which consists of a multiple-protein-binding transactivation domain, a DNA-binding/dimerization domain, and a flexible linker [14]. Consistent with other previous studies, our data confirm that although the C-terminal segment of E2 alone has DNA-binding capacity, lacking its N-terminal portion makes the truncated E2 almost loss it's of promoter repressor activity. Our data indicate that the E2 N-terminal alone works as a transcriptional activator, inducing about 1.5-fold increased promoter activity. However, this positive effect on the promoter is totally abolished in the context of whole E2 protein. The substitution of L118S in the E2 transactivation domain shows no influence on either DNA-binding or promoter activity. The contribution of E2 hinge region to its transcriptional regulator is believed to be not essential [15], as three naturally occurred mutations in this area together do not influence either the E2 DNA-binding or transcriptional activation.

**Figure 7:**
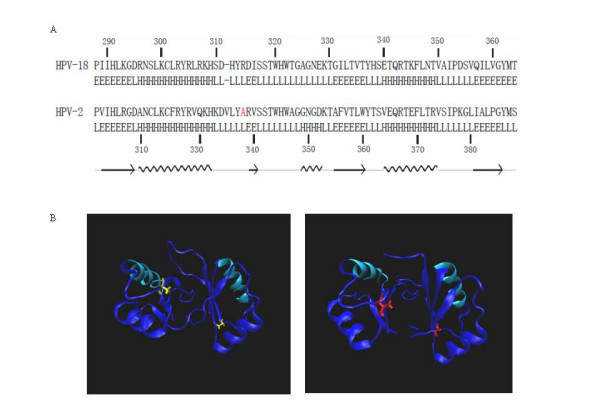
**Molecular modeling of HPV-2 E2 dimeric DNA-binding domain (prototype and mutated forms)**. **A**: Sequence alignment between HPV-2 E2 DNA-Binding domain and HPV-18's (PDB: 1F9F chain: D). There is only one gap between the alignment. In HPV-2 sequence, the A338V mutation is colored red. The secondary structures are derived using the software DSSP. The arrows represent β-strands, and zigzag lines indicate helices. B: The modeled structures of HPV-2 E2 dimeric DNA-Binding domain. Compared to the crystal structure of HPV-18 E2 DNA-Binding domain (PDB: 1F9F), the two helix regions which bind DNA are confirmed (colored cyan). The right structure is mutant form and the residue 338 (Val) is colored red. The modeled structures show that the residue 338 is located in the region of beta barrel

The structural analysis of the C-terminal DBD from several PV E2 proteins, e.g. HPV-16, -18, -31 and bovine papillomavirus (BPV-1), either alone or together with TAD, suggest it to be a tight dimer upon DNA binding [16,17]. The structure of E2 DNA-binding domain is conserved among HPV families [18]. E2 DNA-binding domains of HPV-2 and HPV-18 have 52% identity and there is only one gap between the alignments (Figure 7A). With software Modeller9.5 and NAMD2.6, we have constructed 3D structures of DNA-binding domains of wild-type (338A) and mutant (338 V) E2 proteins using the published crystal structure of HPV-18 E2 DNA-binding domain as the template. The amino acid residue 338 locates in the region of beta barrel that is far away from the helix region that binds to DNA (Figure 7B), indicating that the influence of the mutation on DNA-binding is not due to the direct alteration in the helix region. However, in beta barrel structures the hydrophobic residues are oriented into the interior of the barrel to form a hydrophobic core and the stability of theβ-barrel depends largely on the interaction of the inner hydrophobic amino acid residues. The mutation from Ala to Val at aa 338 increases the hydrophobic property and subsequently stabilizes the dimeric structure of E2, which is possibly responsible for the enhanced DNA binding activities observed in the EMSA.

Previous study has showed that binding of the full-length wild-type BPV-1 E2 protein to the LCR sequences leads to formation of DNA loops and the transcriptional activating domain of E2 is necessary for this loop [19]. Such structure will result in the tissue-specific enhancers shifting closer to the core transcription complex for transcriptional activation [20,21]. Meanwhile, some studies have indicated that binding of the intact E2 to the LCR sequences may spatially prevent the transcriptional machine to active the promoter, which are the main molecular mechanism for E2 transcriptional repression [22,23].

E2 has also been shown to be able to interact with other cellular agents, e.g. Brd4, to regulate its transcriptional activity [24]. The identification of Brd4 as a component in a dominant form of E2 complexes indicates that Brd4 may be the cofactor for HPV E2 repressor function [25]. Apparently, Brd4 recruits E2 that in turn prevents the recruitment of TFIID and pol II to the HPV promoter [26]. Amino acid substitutions within the E2 transactivation domain impaired both the transcriptional activity and binding to Brd4 [27]. Furthermore, Brd4 is a host chromatin adaptor for papillomavirus. The dimerization of the E2 is required for efficient Brd4 binding [28]. The mutation from Ala to Val at aa 338 of HPV-2 E2, which would change the hydrophobicity and/or tertiary structure of E2, will lead to a modification of its interaction with the chromatin, and thus, modulates its transcriptional regulation activity. Although our data highlight a close correlation between the increased activity for DNA-binding and the enhanced activity for viral early promoters of the mutated E2 protein, the exact mechanism remains unclear.

Our data indicate that the DNA-binding capacity of the C-terminal fragment of E2 is stronger than those with the hinge region, and much stronger than the full-length E2. Earlier study has found that besides the full-length E2, bovine papillomavirus (BPV) E2 ORF also encodes two other E2 peptides, E2-TR and E8/E2 proteins [29]. These shorter E2 proteins contain the DNA binding and dimerization domains of the C-terminus and hinge region, but lack the transactivation domain. Relative abundances of the truncated E2 proteins have been observed in BPV transformed cells (the molar ratio of E2:E2-TR:E8/E2 is 1:10:3) [30]. Expression of HPV-31 E8E2C protein has been reported to be able to inhibit HeLa cell growth [31]. However, the transcriptional profiles of other HPV E2 ORFs, regardless in benign or malignant cells, are rarely addressed. The fact that the C-terminus E2 binds DNA stronger suggests that it is more competitive than the full-length E2 in the cells.

Our study provides the evidence that HPV-2 E2, regardless of wild-type or mutant (A338V), induces the similar biological effectiveness under the controls of the homologous and heterologous HPV LCRs. This suggests that E2 protein may induce same regulative activity on the viral early promoters from different HPVs with similar upstream components. Although there are more than 100 genotypes of HPVs involving in various human benign or malignant proliferating diseases, the sequences of viral genomes are relatively conservative. Hence, the effectiveness of HPV-2 E2 may represent a common property of HPVs' E2 proteins.

In addition to the role in regulating viral transcription, HPV E2 protein involves in enhancing E1-dependent viral DNA replication and genome maintenance. In HPV genomes the viral DNA replication initiation site co-localizes with the viral transcription region. However, the regulative function of E2 in viral DNA replication is far from understood compared with its role in transcriptional regulation. Although the point mutations in TAD and in hinge region within this E2 mutant do not affect DNA-binding and transcriptional regulation, their influence on viral genome replication cannot be excluded. Sequences analyses of this variant HPV-2 strain have also identified several point mutations in its E1 ORF. Further studies of viral genome replication will help explore the inconvenient reason of such huge verrucae vulgaris.

## Conclusions

Our study provides evidence that HPV-2 E2 with Ala to Val mutation at aa 338 is critical for E2 DNA-binding and its transcriptional regulation. The binding abilities of E2 proteins with A338V to either an artificial probe containing with two E2 binding sites or HPV-2 and HPV-16 promoter-proximal LCR sequences were significantly stronger than that of HPV-2 prototype E2. Furthermore, co-expression of the E2 constructs containing A338V mutation induces higher activities on heterologous HPV-16 early promoter P97 than that of prototype E2.

## Methods

### Plasmids construction

Mammalian expression plasmid pcDNA-E2-proto containing the whole E2 sequences of HPV-2 prototype, plasmid pcDNA-E2-Mut containing the whole E2 sequences of isolate 1 and CAT reporter plasmid pCAT-LCR(L) containing HPV-2 prototype LCR sequence (from nt 6934 to 134) were generated previously [13]. The plasmid pCAT-LCR-HPV16 containing HPV-16 LCR was generated previously [32]. Sited-direction mutation PCR was performed using pcDNA-E2-proto or pcDNA-E2-Mut as the templates to generate various E2 sequences with one to four point mutations. Table 1 summarized the primers used in PCR, in which the primers amplifying whole E2 ORF (from nt 2685 to 3860), E2 N-segment (from nt 2685 to 3279) and E2 C-segment (from nt 3618 to 3860) contained a Hind III site in the up-steam and Bam HI site in the down-steam primers.

**Table 1:** The primers for site-directed mutation PCR

Primers	The sequence of the primers	Enzyme site
E2-up	5'AAGCTTATGGAAACACTGGCGAACCGT3'	Hind III

E2-down	5'GGATCCTTATACAAATGCAGACATATACCC3'	BamHI

E2N-down	5'GGATCCTTATGATTCTGCTGAGGC3'	BamHI

E2C-up	5'CTTAAGGTGGCTGGGACTGTTATTCAC3'	Hind III

118-up	5'CAGTACTAGTGAAATTTGATGGCAGC3'	\

118-down	5'GCTGCCATCAAATTTCACTAGTACTG3'	\

235-up	5'CAGAACCAACAGGAGCAGGAAG3'	\

235-down	5'GTGGAGGACACCCTGGCATAC3'	\

338-up	5'GTATGCCAGGGTGTCCTCCAC3'	\

338-down	5'GTGGAGGACACCCTGGCATAC3'	\

m338-up	5'GTATGTCAGGGTGTCCTCCAC3'	\

m338-down	5'GTGGAGGACACCCTGACATAC3'	\

E2-P-up	5'GGATCCATGGAAACACTGGCGAACCGT3'	BamHI

E2N-P-down	5'GAATTCTTATGATTCTGCTGAGGC3'	EcoRI

E2C-P-up	5'GGATCCGTGGCTGGGACTGTTATTCAC3'	BamHI

E2-P-down	5'GAATTCTTATACAAATGCAGACATATACCC3'	EcoRI

E2-P-117-up	5'GGATCCTACATCCGCATCTGTGTCTAG3'	BamHI

PCR amplification was performed in 25 μl of a reaction mixture containing 2.5 U of Taq DNA polymerase (TaKaRa, Dalian, China), 20 mM dNTP, 150 ng of each mixture of HPV-2 E2 specific primers at the cycle condition of denaturing at 94°C for 30 s, annealing at 58°C for 30 s, extending at 72°C for 1 min, totally 30 cycles, respectively. Briefly, to construct the E2-sequence containing single point-mutation including the mutants of L118S, S235P or A338V, two separated PCR amplifications were conducted using pcDNA-E2-proto as the templates, with the primer mixture of E2-up with 118-down, 235-down or m338-down, and the mixture of E2-down with 118-up, 235-up or m338-up, respectively. After purified, two individual PCR products were mixed and annealed, and the sequence covering whole E2 ORF of each mutant was constructed by another PCR amplification with primers E2-up and E2-down, generating E2-L118S, E2-S235P and E2-A338V, respectively. To generate E2-sequence containing two point-mutations of L118S and S235P, two separated PCRs were conducted based on the sequence of E2-L118S, with the primes E2-up and 235-down, as well as 235-up and E2-down, respectively. The whole E2 sequence of this mutant was obtained with the same protocol above, generating E2-L118S/S235P. To construct E2-sequence containing other four mutations except A338V, the PCR reactions were separately performed using E2-Mut sequence as the template, with the primer E2-up and 338-down, as well as 338-up and E2-down. The whole E2 sequence was obtained based on the protocol above, generating E2-Mut (-). E2 N-segment (from nt 2685 to 3279) and E2 C-segment (from nt 3618 to 3860) were generated by PCR with respective primer mixtures. The generated E2 sequences were cloned into plasmid pMD18-T. After verified with sequencing assays, various E2 segments were released from cloning vectors and subcloned into pcDNA3.1, generating mammalian expressing recombinant plasmids pcDNA-E2 (Figure 1A).

To construct the different HPV-2 E2 prokaryotic expressing plasmids, including E2 of prototype (E2), E2 of isolate 1 (E2-Mut) and various mutated E2, three lengths of E2 sequences, including the full-length E2 ORF (from aa 1 to 391, FL), the sequence starting from hinge region to the end (from aa 197 to 391, HC) and C-terminal segment (from aa 311 to 391, C), were generated by PCR technique with different primer mixtures, using individual pcDNA-E2 as the templates. The PCR products were cloned into plasmid pMD18-T and subcloned into a (GST) expression vector pGEX-2 T, generating various plasmids pGST-E2 (Figure 2A).

### Cell line, transfection and CAT assay

The human cervical cancer cell lines HeLa were maintained in Dulbecco's modified Eagle's medium (Invitrogen) with 10% fetal calf serum (HyClone). C33A and SiHa cell lines were maintained in ATCC-formulated Eagle's Minimum Essential Medium (Catalog No.30-2003) with 10% fetal calf serum. Cells were plated into 60 mm 6-well plates (Falcon, Japan) one day before transfection. 2 μg of plasmid pCAT-LCR were transfected with Lipofectamine 2000 transfection reagent (Invitrogen, USA), together with 1 μg of pCMV-β-galactosidase as internal control. To evaluate the effectiveness of E2 protein on the promoter activity, various E2 expression plasmids (500 ng) were co-transfected into cells. Cells were harvested at 48 h after transfection.

CAT expressions were measured quantitatively using a CAT ELISA kit (Roche, Switzerland), according to the instruction manual. The expression of β-galactosidase activity was determined using O-nitrophenyl-β-D-galactopyranoside (ONPG) as a colorimetric substrate. HPV-2 promoter activities were determined by calculating the rates of CAT and β-galactosidase values. Each experiment was independently performed for three to five times.

### Western blots

HeLa and C33A cells transfected with 500 ng various E2 expressing plasmids, together with or without 2 μg pCAT-LCR, were harvested 24, 48 and 72 h post-transfection. Cells were pelleted by short centrifugation and suspended in the lysis buffer (10 mM Tris-HCl, pH 7.8, 0.5% sodiumdeodycholate, 0.5% Nonidet P-40, 100 mM NaCl, 10 mM EDTA), supplemented with complete proteasomal inhibitor mixture. Cell lysates were separated by 15% SDS-PAGE and electro-transferred onto nitrocellulose membranes. After blocking with 5% nonfat-dried milk in PBS (phosphate buffered saline, pH 7.6) overnight at 4°C, the membranes were incubated with 1:1,000 HPV E2 specific monoclonal antibody at room temperature (RT). After washing with PBST (phosphate buffered saline, pH 7.6, containing 0.05% Tween-20), the membranes were incubated with 1:5,000 horseradish peroxidase (HRP)-conjugated anti-mouse antibody. The E2 protein signals were visualized by ECL kit (PE Applied Biosystems, USA). To reuse the blotted membrane, the developed membrane was treated in the Restore Western Blot Stripping Buffer (Thermo, USA) for 10 min at RT. 1:1,000 mAb anti-human β-actin (Santa Cruz, USA) and HRP-conjugated anti-mouse antibody were used to identify β-actin protein. ECL kit was used to visualize the signals.

### Expression and purification of E2 proteins

The recombinant prokaryotic proteins tagged with GST were bacterially expressed in *E. coli *BL21 and purified with Glutathione Sepharose 4B Agarose (Pharmacia, USA) according the protocol described in our previous study [33]. The purities of the purified proteins were verified by 15% SDS-PAGE.

### Electrophoretic mobility shift assays (EMSA)

For EMSA, single DNA oligos labeled with biotin that covered two E2 binding sites (HPV-E2BS) ACCGAAAACGGTCAGACCGAATTCGGTTGT3' and 5'ACAACCgAATTCGGTCTGACCGTTTTCGGTCACAC3') and HPV-16 (5'GGCGTAACCGAAATCGGTTGAACCGAAACCGGTT3' and 5'AACCGGTTTCGGTTCAACCGATTTCGGTTACGCC3') were synthesized based on the sequences in GenBank. After denaturing by heating, two signal DNA oligos were annealed at RT to a double-stranded DNA probe. 12.5 to 250 fM biotin-labeled oligonucleotide probes were mixed with various amounts of different E2 proteins in 20 μl binding buffer (10 mM Tris, 50 mM KCl, 1 mM DTT, pH 7.5) at RT for 20 min. For competition experiments, biotin-labeled oligo HPV-E2BS was competed with 50-, 100- and 500-fold excess of unlabeled homologous oligo or 500-fold excess of unlabeled heterologous oligo (T7 promoter double-stranded sequences). For supershift EMSA, 7 μg recombinant HPV-2 E2-FL was incubated with mAb against HPV-E2 at RT for 30 min, prior to mixing with biotin-labeled oligo HPV-E2BS. DNA-protein complexes were separated from unbound probe in a 6.5% non-denaturing polyacrylamide gel and visualized by LightShift^® ^Chemiluminescent EMSA Kit (Pierce, USA) according to the manufacturer's instruction. Quantitative analysis of images was carried out using computer-assisted software Image Total Tech (Pharmacia, USA). The image was scanned with Typhoon (Pharmacia, USA), digitalized and saved as TIF format.

### Structure analysis

The 3D structures of prototype and mutated HPV-2 E2 DNA-binding domain were modeled based on the existed crystal structure of HPV E2 with the help of software Modeller9.5 and NAMD2.6 (optimize the structure by energy minimization). The quaternary structure of HPV-18 (PDB: 1F9F) was choose as the template.

### Molecular modeling

Molecular models of prototype and mutated HPV-2 E2 DNA-binding domain were constructed using the homology modeling software Modeller v9.5 [8]. The closely related structure of HPV-18 E2 DNA-binding domain (PDB: 1F9F) [34] was used as the template. The resulting structure files were subjected to energy minimization using NAMD2.6 [18].

## Abbreviations

HPV: The human papillomavirus; EMSA: Electrophoresis mobility shift assays; ORFs: Open reading frames; LCR: Long control region; URRs: Upstream regulatory regions; TAD: Transcriptional activation domain; DBD: DNA-binding/dimerization domain; mAb: monoclonal antibody; E2BS: E2 protein binding sites; GST: Glutathione S-transferase; PV: Papillomavirus; BPV: Bovine papillomavirus; ONPG: O-nitrophenyl-β-D-galactopyranoside; RT: Room temperature; HRP: Horseradish peroxidase.

## Competing interests

The authors declare that they have no competing interests.

## Authors' contributions

CG, MMP and XPD designed the overall study. CG and JHY carried out the cell culture, transfections and CAT assays. CG also performed the data analysis and interpretation. XPD wrote the initial draft of the manuscript. MMP, XLL and QS participated in expressions of recombinant proteins, and carried out the experiments related to EMSAs. YJL and CT designed the primers of PCRs, built the plasmid constructions used in this study. LQT contributed to the bioinformatic analyses. YKY and GXF coordinated the study and drafted the final version of the manuscript. All authors read and approved the final manuscript.

## References

[B1] de Villiers EM Fauquet C Broker TR Bernard HU zur Hausen H Classification of papillomaviruses Virol 2004 324 17 27 10.1016/j.virol.2004.03.033 15183049

[B2] McBride AA Romanczuk H Howley PM The papillomavirus E2 regulatory proteins J Biol Chem 1991 266 18411 18414 1655748

[B3] Romanczuk H Thierry F Howley PM Mutational analysis of cis elements involved in E2 modulation of human papillomavirus type 16 P97 and type 18 P105 promoters J Virol 1990 64 2849 2859 2159546 10.1128/jvi.64.6.2849-2859.1990 PMC249467

[B4] Steger G Corbach S Dose-dependent regulation of the early promoter of human papillomavirus type 18 by the viral E2 protein J Virol 1997 71 50 58 8985322 10.1128/jvi.71.1.50-58.1997 PMC191023

[B5] Hegde RS Androphy EJ Crystal structure of the E2 DNA-binding domain from human papillomavirus type 16: implications for its DNA binding-site selection mechanism J Mol Biol 1998 284 1479 1489 10.1006/jmbi.1998.2260 9878365

[B6] Soeda E Ferran MC Baker CC McBride AA Repression of HPV16 early region transcription by the E2 protein Virol 2006 351 29 41 10.1016/j.virol.2006.03.016 16624362

[B7] De la Cruz-Hernández E García-Carrancá A Mohar-Betancourt A Dueñas-González A Contreras-Paredes A Differential splicing of E6 within human papillomavirus type 18 variants and functional consequences J Gen Virol 2005 86 2459 2468 10.1099/vir.0.80945-0 16099904

[B8] Sali A Blundell TL Comparative protein modelling by satisfaction of spatial restraints J Mol Biol 1993 234 779 815 10.1006/jmbi.1993.1626 8254673

[B9] Ptashne M Regulation of transcription: from lambda to eukaryotes Trends Biochem Sci 2005 30 275 279 10.1016/j.tibs.2005.04.003 15950866

[B10] Cricca M Venturoli S Leo E Costa S Musiani M Disruption of HPV 16 E1 and E2 genes in precancerous cervical lesions J Virol Methods 2009 158 180 183 10.1016/j.jviromet.2009.01.005 19187786

[B11] Wang C Wang W Lei YJ Wang JY Dong XP Multiple huge cutaneous horns overlying verrucae vulgaris induced by human papillomavirus type 2: a case report Br J Dermatol 2007 156 760 762 10.1111/j.1365-2133.2006.07734.x 17493077

[B12] Wang C Xu S Chen C Tong X Liang Y Detection of HPV-2 and identification of novel mutations by whole genome sequencing from biopsies of two patients with multiple cutaneous horns J Clin Virol 2007 39 34 42 10.1016/j.jcv.2007.01.002 17368088

[B13] Lei YJ Wang C Gao C Jiang HY Chen JM HPV-2 isolates from patients with huge verrucae vulgaris possess stronger promoter activities Intervirol 2007 50 353 360 10.1159/000107706 17728546

[B14] McBride AA Byrne JC Howley PM E2 polypeptides encoded by bovine papillomavirus I form dimers through the carboxyl-terminal DNA binding domain: Transactivation is mediated through the conserved amino-terminal domain Proc Natl Acad Sci USA 1989 86 510 514 10.1073/pnas.86.2.510 2536165 PMC286500

[B15] Giri I Yaniv M Structural and mutational analysis of E2 trans-activating proteins of papillomaviruses reveals three distinct functional domains EMBO J 1988 7 2823 2829 2846285 10.1002/j.1460-2075.1988.tb03138.x PMC457074

[B16] Cicero DO Nadra AD Eliseo T Paci M dePrat-Gray G Structural and thermodynamic basis for the enhanced transcriptional control by the human papillomavirus strain-16 E2 protein Biochem 2006 45 6551 6560 10.1021/bi060123h 16716065

[B17] Hegde RS Grossman SR Laimins LA Sigler PB Crystal structure at 1.7 Å of the bovine papillomavirus-1 E2 DNA-binding domain bound to its DNA target Nature 1992 359 505 512 10.1038/359505a0 1328886

[B18] Kim SS Tam JK Wang AF Hegde RS The structural basis of DNA target discrimination by papillomavirus E2 proteins J Biol Chem 2000 275 31245 31254 10906136 10.1074/jbc.M004541200

[B19] Knight JD Li R Botcham M The activation domain of the bovine papillomavirus E2 protein mediates association of DNA-bound dimers to form DNA loops Proc Natl Acad Sci USA 1991 88 3204 3208 10.1073/pnas.88.8.3204 1849647 PMC51414

[B20] Hernandez-Ramon EE Burns JE Zhang W Walker HF Allen S Dimerization of the human papillomavirus type 16 E2 N terminus results in DNA looping within the upstream regulatory region J Virol 2008 82 4853 4861 10.1128/JVI.02388-07 18337573 PMC2346762

[B21] O'Connor M Chan SY Bernard HU Myers G, Bernard H-U, Delius H, Baker C, Icenogel J, Halpern A, Wheeler C Transcription factor binding sites in the LCR of genital HPVs, p.III-21-III-46 Human papillomavirus 1995 Los Alamos: Los Alamos National Laboratory

[B22] Dong G Broke TR Chow LT Human papillomavirus type 11 E2 proteins repress the homologous E6 promoter by interfering with the binding of host transcription factors to adjacent elements J Virol 1994 68 1115 1127 8289341 10.1128/jvi.68.2.1115-1127.1994 PMC236550

[B23] Dostatni N Lambert PF Sousa R Ham J Howley PM The functional BPV-1 E2 transactivating protein can act as a repressor by preventing formulation of the initiation complex Genes Dev 1991 5 1657 1671 10.1101/gad.5.9.1657 1653173

[B24] Wu SY Chiang CM The double bromodomain-containing chromatin adaptor Brd4 and transcriptional regulation J Biol Chem 2007 282 13141 13145 10.1074/jbc.R700001200 17329240

[B25] Wu SY Lee AY Hou SY Kemper JK Erdjument-Bromage H Brd4 links chromatin targeting to HPV transcriptional silencing Genes Dev 2006 20 2383 2396 10.1101/gad.1448206 16921027 PMC1560413

[B26] Yan J Li Q Lievens S Tavernier J You J Abrogation of the Brd4-positive transcription elongation factor B complex by papillomavirus E2 protein contributes to viral oncogene repression J Virol 2010 84 76 87 10.1128/JVI.01647-09 19846528 PMC2798453

[B27] Sénéchal H Poirier GG Coulombe B Laimins LA Archambault J Amino acid substitutions that specifically impair the transcriptional activity of papillomavirus E2 affect binding to the long isoform of Brd4 Virology 2007 358 10 7 10.1016/j.virol.2006.08.035 17023018

[B28] Cardenas-Mora J Spindler JE Jang MK McBride AA Dimerization of the papillomavirus E2 protein is required for efficient mitotic chromosome association and Brd4 binding J Virol 2008 82 7298 7305 10.1128/JVI.00772-08 18495759 PMC2493320

[B29] Lambert PF Spalholz BA Howley PM A transcriptional repressor encoded by BPV-1 shares a common carboxy-terminal domain with the E2 transactivator Cel 1997 50 69 78 10.1016/0092-8674(87)90663-5 3036366

[B30] Lambert PF Hubbert NL Howley PM Schiller JT Genetic assignment of multiple E2 gene products in bovine papillomavirus-transformed cells J Virol 1989 63 3151 3154 2542621 10.1128/jvi.63.7.3151-3154.1989 PMC250873

[B31] Stubenrauch F Straub E Fertey J Iftner T The E8 repression domain can replace the E2 transactivation domain for growth inhibition of HeLa cells by papillomavirus E2 proteins Int J Cancer 2007 121 2284 2292 10.1002/ijc.22907 17583574

[B32] May M Dong XP Beyer-Finkler E Stubenrauch F Fuchs PG The E6/E7 promoter of extrachromosomal HPV16 DNA in cervical cancers escapes from cellular repression by mutation of target sequences for YY1 EMBO J 1994 13 1460 1466 8137827 10.1002/j.1460-2075.1994.tb06400.x PMC394965

[B33] Dong CF Shi S Wang XF An R Li P The N-terminus of PrP is responsible for interacting with tubulin and fCJD related PrP mutants possess stronger inhibitive effect on microtubule assembly in vitro Arch Biochem Biophys 2007 470 83 92 18037369 10.1016/j.abb.2007.11.007

[B34] Phillips JC Braun R Wang W Gumbart J Tajkhorshid E Scalable molecular dynamics with NAMD J Comput Chem 2005 26 1781 1802 10.1002/jcc.20289 16222654 PMC2486339

